# Predisposing factors for deep sternal wound infection after cardiac surgery

**DOI:** 10.1186/cc14090

**Published:** 2015-03-16

**Authors:** F Ampatzidou, M Sileli, A Madesis, K Antoniou, A Baddur, G Kechagioglou, T Asteri, G Drossos

**Affiliations:** 1 General Hospital G. Papanikolaou Thessaloniki, Greece

## Introduction

The aim of our study was to investigate perioperative risk factors associated with deep sternal wound infections in complicated cardiac surgery.

## Methods

A total of 1,017 patients underwent cardiac surgery in a 2-year period. We investigate the correlation between deep sternal wound infection with the following 14 preoperative characteristics and perioperative parameters: age >75, female gender, diabetes mellitus (DM), insulin dependence, body mass index (BMI) >30, current smokers, COPD, cardiopulmonary bypass time (CBP) >120 minutes, use of steroids, emergency operation, prolonged mechanical ventilation (>48 hours), reintubation, transfusion with more than 3 units of red blood cells, and the postoperative use of non-invasive ventilation (NIV). The chi-square test was used for statistical analysis.

## Results

A total of 35 patients (3.44%) were complicated by deep sternal wound infections. No statistical correlation was found with age >75, gender, DM, BMI >30, steroids, emergent operation, prolonged ventilation, CBP time >120 minutes, reintubation and NIV. Factors with statistical significant correlation are presented in Table 1.

**Table 1:** 

	Sternal infection	*P *value
Insulin	9/85	0.001
Current smoker	4/272	0.037
COPD	11/197	<0.001
Transfusion >3	19/302	0.001

## Conclusion

Postoperative deep sternal wound infections have statistical significant correlation with the following parameters: transfusion with >3 red blood cell units, history of COPD, insulin dependence and when the patient is a current smoker. Also there is a tendency for correlation with CBP time >120 minutes (*P *= 0.056).

## References

[B1] Diez C Risk factors for mediastinitis after cardiac surgery - a retrospective analysis of 1700 patients J Cardiothor Surg 2007 2 23 10.1186/1749-8090-2-23 PMC1891287 17511885

[B2] Schimmer C Prevention of sternal dehiscence and infection in high-risk patients: a prospective randomized multicenter trial Ann Thorac Surg 2008 86 1897 904 10.1016/j.athoracsur.2008.08.071 19022005

